# Exposure to long wavelength light that improves aged mitochondrial function shifts acute cytokine expression in serum and the retina

**DOI:** 10.1371/journal.pone.0284172

**Published:** 2023-07-21

**Authors:** Harpreet Shinhmar, Chris Hogg, Glen Jeffery

**Affiliations:** Institute of Ophthalmology, University College London, London, United Kingdom; University of Florida, UNITED STATES

## Abstract

Aged mitochondrial function can be improved with long wavelength light exposure. This reduces cellular markers of inflammation and can improve system function from fly through to human. We have previously shown that with age there are increases in cytokine expression in mouse serum. Here, we ask what impact 670nm light has on this expression using a 40 cytokine array in blood serum and retina in C57Bl6 mice. 670nm exposure was delivered daily for a week in 12 month old mice. This shifted patterns of cytokine expression in both serum and retina inducing a selective increase. In serum examples of significant increases were found in IL (interleukins) 1α, IL-7, 10, 16, 17 along with TNF-α and CXCL (chemokines) 9 and 10. In retina the increases were again mainly in some IL’s and CXCL’s. A few cytokines were reduced by light exposure. Changes in serum cytokines implies that long wavelengths impact systemically even to unexposed tissues deep in the body. In the context of wider literature, increased cytokine expression may be protective. However, their upregulation by light merits further analysis as cytokines upregulation can also be negative and there are probably complex patterns of interaction in the dynamics of their expression.

## Introduction

Mitochondria regulate metabolism and the pace of ageing. When their membrane potential declines adenosine triphosphate (ATP) production is compromised reducing available cellular energy. This is often associated with progressive increases in reactive oxygen species (ROS) that further undermines cell function [1]. However, long wavelength light (650-900nm) can reverse many of these features in ageing and disease, particularly in the CNS with its high metabolic demand and mitochondrial dependence [2].

Exposure to longer wavelengths has been widely shown to have therapeutic value in the CNS. In invertebrates it extends lifespan and improves aged motor skills, cognition and visual function [3–6]. In mammals it has similar impact and also reduces cellular markers of inflammation and the pace of age related cell loss [7–9]. Its application is now extended to humans where aged visual function is significantly improved [10].

There is limited evidence that longer wavelength exposure also impacts on immunity [11–13]. However, this remains relatively unexplored. A key question here relates to whether changes in immunity induced by longer wavelengths can also be found in serum. If this were the case, then it may imply that the impact of such lights can act systemically. There has been evidence for this when longer wavelengths have been targeted at distal regions of the body and have had positive impacts on the retina [13]. But the mechanism for this has not been revealed.

The absence of data on the interactions between immunity and longer wavelengths is problematic for the development of therapies based on their use. We have previously shown in mouse serum that ageing is associated with a general increase in cytokine expression [14]. Here we expose aged mice to a commonly used long wavelength, 670nm, and assess its impact on the cytokine expression in blood serum and retina. The hypothesis is that consistent with improved mitochondrial function, there will be a decline in the patterns of cytokine expression following 670nm light exposure. This proved not to be the case.

## Methods

### Animals

Investigations were performed under a UK Home Office Project License (PPL 94/5839) in accordance with UK and EU regulation and approved by the UCL Animal Welfare and Ethical Review Body. All methods were carried out in compliance with ARRIVE guidelines.

A total of 12 old male C57Bl6 mice at 12 months of age were used. The investigation consisted of an age-matched control group housed under identical conditions, and an experimental group who were exposed to long-wavelength light at 670nm (40mW/cm^2^, CH Electronics UK) daily at 10am for 15 minutes lasting 1 week. Mice were exposed to light within their cages [15], and free to roam thereby reducing the amount of stress to the animals. Samples of blood serum and retina from individual mice in both control (N = 6) and 670nm light exposed (N = 6) groups were analysed to allow for statistical analysis.

All mice were killed by cervical dislocation. Eyes were rapidly removed, and the retina extracted on ice and processed as tissue lysates as below. Bloods were taken via cardiac puncture prior to cervical dislocation.

### Blood serum collection

To avoid false cytokine readings and minimize stress all animals were acclimatized to the room and person for at least 1 hour prior to time of death. Mice were deeply anaesthetized to allow for open chest cardiac puncture. The amount of blood collected with very little needle movement as possible to avoid hemolysis of samples was limited to 0.5ml per animal, as this would commonly yield ∼0.2ml of serum. Blood was rapidly harvested into tubes and allowed to coagulate on ice for approximately 20 minutes. After the blood had coagulated for 20 minutes the tubes were then centrifuged at 2000 x g for 15 minutes and the serum that was creamy white was transferred to a new tube. Protein concentration was calculated using a BCA Assay kit (Thermo Scientific). As advised by the manufacturer’s protocol (Proteome Profiler, R&D Systems, Minneapolis, USA), 150μl of serum per group was added to each cytokine array membrane from both pooled samples and individual mouse samples.

### Tissue lysates

Retinal tissue was prepared according to the manufacturer’s protocol (Proteome Profiler, R&D Systems, Minneapolis, USA). The retinae were homogenized by hand in PBS containing protease inhibitors (Sigma, UK). Samples were then snap frozen at ≤ -70°C with the addition of Triton X-100 to a final concentration of 1%. Lysates were thawed and centrifuged at 10,000 x g for 10 minutes to remove cellular debris and the resulting supernatant collected. Protein concentration was calculated using a BCA Assay (Thermo Scientific). As advised by the manufacturer’s protocol 200μg of protein from retinal lysates was added to each cytokine array membrane from both pooled samples and individual mouse samples.

### Cytokines

Cytokine levels were assessed using the Proteome Profiler Mouse Cytokine Array Panel A (R&D Systems, Minneapolis, USA) according to the protocol below. Array nitrocellulose membranes were incubated for 1 hour on a rocking platform with Array buffer 6, a buffered protein base with preservatives, which served as a blocking buffer. Whilst blocking, the samples (blood serum and retina) were incubated for 1 hour with Array buffer 4, a buffered protein base with preservatives, and a biotinylated antibody cocktail. The membranes were then incubated overnight on the rocking platform with the sample/antibody mixture. The following day the membranes were washed several times with a wash buffer, a solution of buffered surfactant. The arrays were then incubated for 30 minutes with Streptavidin-HRP (Streptavidin conjugated to horseradish-peroxidase) on a rocking platform. The membranes were then washed again several times with wash buffer. Any excess liquid was drained off the membranes before a Chemi reagent mix, made up of stabilised hydrogen peroxidase and luminol, was applied and incubated for 1 minute. Any excess reagent was removed before the membranes were fixed in an autoradiography film cassette and exposed to an X-ray film. Protein Array Analyzer for Image J was used to quantify and determine spot density from the X-ray film.

### Statistical analysis

All results were drawn up and analysed using GraphPad Prism 6 (GraphPad, San Diego, USA) with a 2-way ANOVA used to determine the overall statistical significance and a Mann-Whitney U statistical test for individual cytokine comparisons. Any significant results where p≤0.05 are denoted in the figure legends and tables.

## Results

Cytokine expressions were examined from blood serum and the retina in 12-month-old mice exposed to 670nm light for 1 week and compared against age-matched unexposed controls.

**Fig 1:**
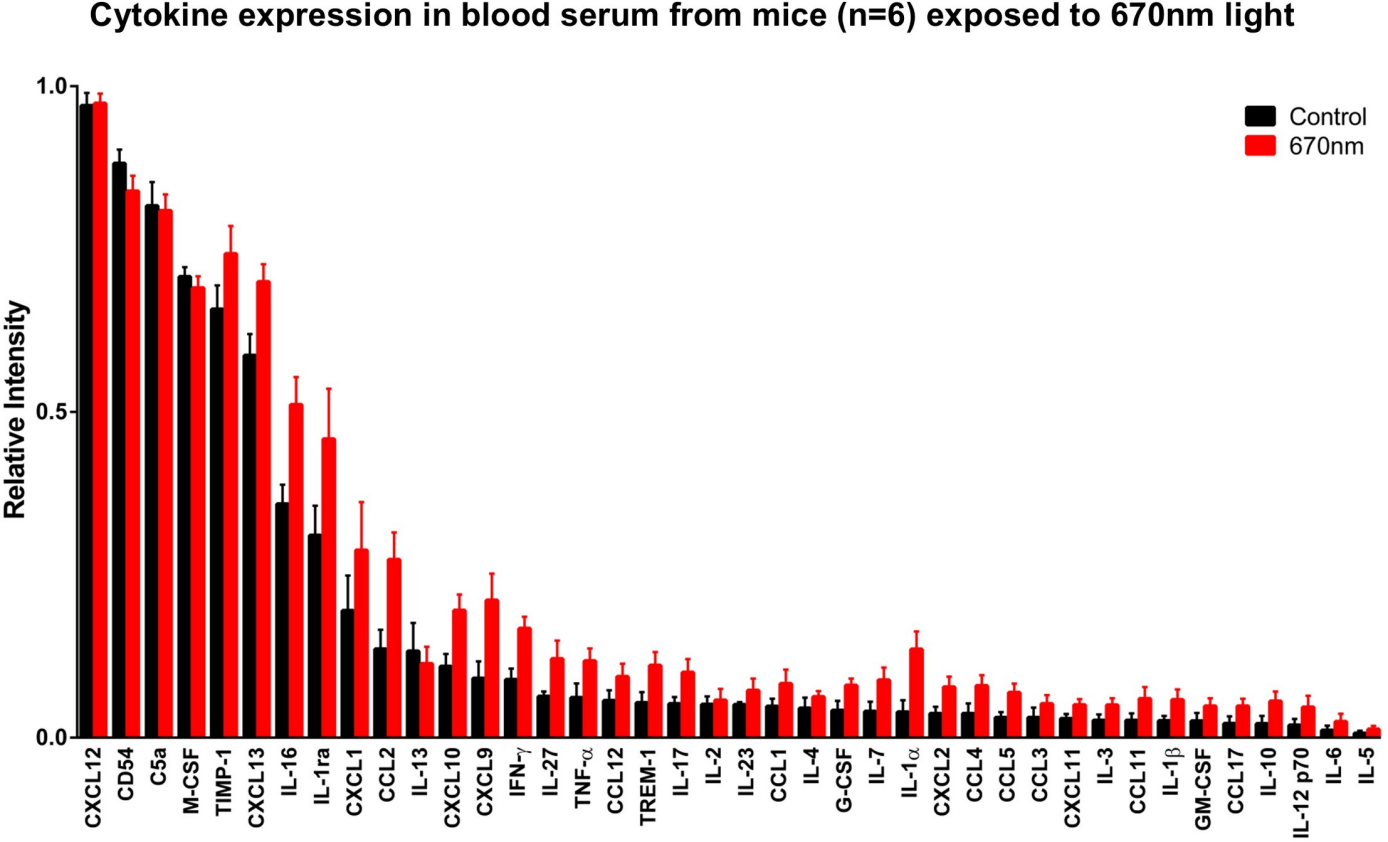
Cytokine expression serum. Cytokine expression from blood serum of 12-month-old C57Bl6 mice (N = 6) following exposure with 670nm light (N = 6. Red) for 1 week compared to aged match controls (N = 6. Black). Cytokines are ordered with the highest expressing on the left and the lowest expressing on the right. Overall, across all 40 cytokines there was a 20% increase in cytokines from 670nm light exposed animals. A 2-way ANOVA revealed a significance of p≤0.0001. Individual cytokine comparisons are presented in Table 1. Error bars = SEM.

Cytokines in blood serum (Fig 1) are ordered according to expression in control mice, with those highest in concentration on the left and those lowest in concentration on the right. All 40 cytokines were expressed in both groups, with a statistically significant (p≤0.0001, 2-way ANOVA) increase of 20% in 670nm light exposed animals. Multiple comparisons of individual cytokines from Fig 1 are displayed in Table 1. Here they are ordered from highest to lowest percentage differences between both groups. There were large differences in both the interleukin and chemokine families, with no apparent pattern as to whether they have an anti-inflammatory or pro-inflammatory roles.

**Table 1:** Individual cytokine comparison in blood serum between 12-month-old C57Bl6 mice exposed to 670nm light for 1 week compared to age matched controls, represented in Fig 1.

Target	P-Value	Significance	% Difference
IL-1α	0.013	*	71
IL-10	0.0325	*	62
IL-12 p70	0.119	ns	60
CXCL9	0.0465	*	57
CCL11	0.119	ns	56
CCL17	0.0898	ns	56
CCL5	0.0465	*	55
IL-1β	0.0898	ns	55
IL-7	0.0465	*	54
IL-6	0.1526	ns	54
CCL4	0.0206	*	54
CXCL2	0.0325	*	52
TREM-1	0.0206	*	52
CCL2	0.0206	*	50
IL-17	0.0325	*	48
TNF-α	0.0325	*	48
G-CSF	0.0206	*	48
IL-27	0.0898	ns	48
IL-5	0.1645	ns	48
GM-CSF	0.066	ns	47
IL-3	0.066	ns	47
IFN-γ	0.013	*	47
CXCL10	0.0076	**	44
CCL1	0.1937	ns	42
CXCL11	0.0325	*	42
CCL3	0.119	ns	41
CCL12	0.0898	ns	39
CXCL1	0.1937	ns	32
IL-1ra	0.1526	ns	32
IL-23	0.2857	ns	30
IL-16	0.0152	*	30
IL-4	0.119	ns	28
CXCL13	0.026	*	16
IL-13	0.4491	ns	-14
IL-2	0.3939	ns	12
TIMP-1	0.119	ns	11
CD54	0.0898	ns	-5
M-CSF	0.2857	ns	-2
C5a	0.4491	ns	-1
CXCL12	0.4491	ns	0

The final column represents a heat map of the relative percentage increase or decrease values in expression of each cytokine with red indicating a relatively large change and green a relatively small change. Mann-Whitney U statistical test

*p≤0.05

**p≤0.01, ns = non-significant.

**Fig 2:**
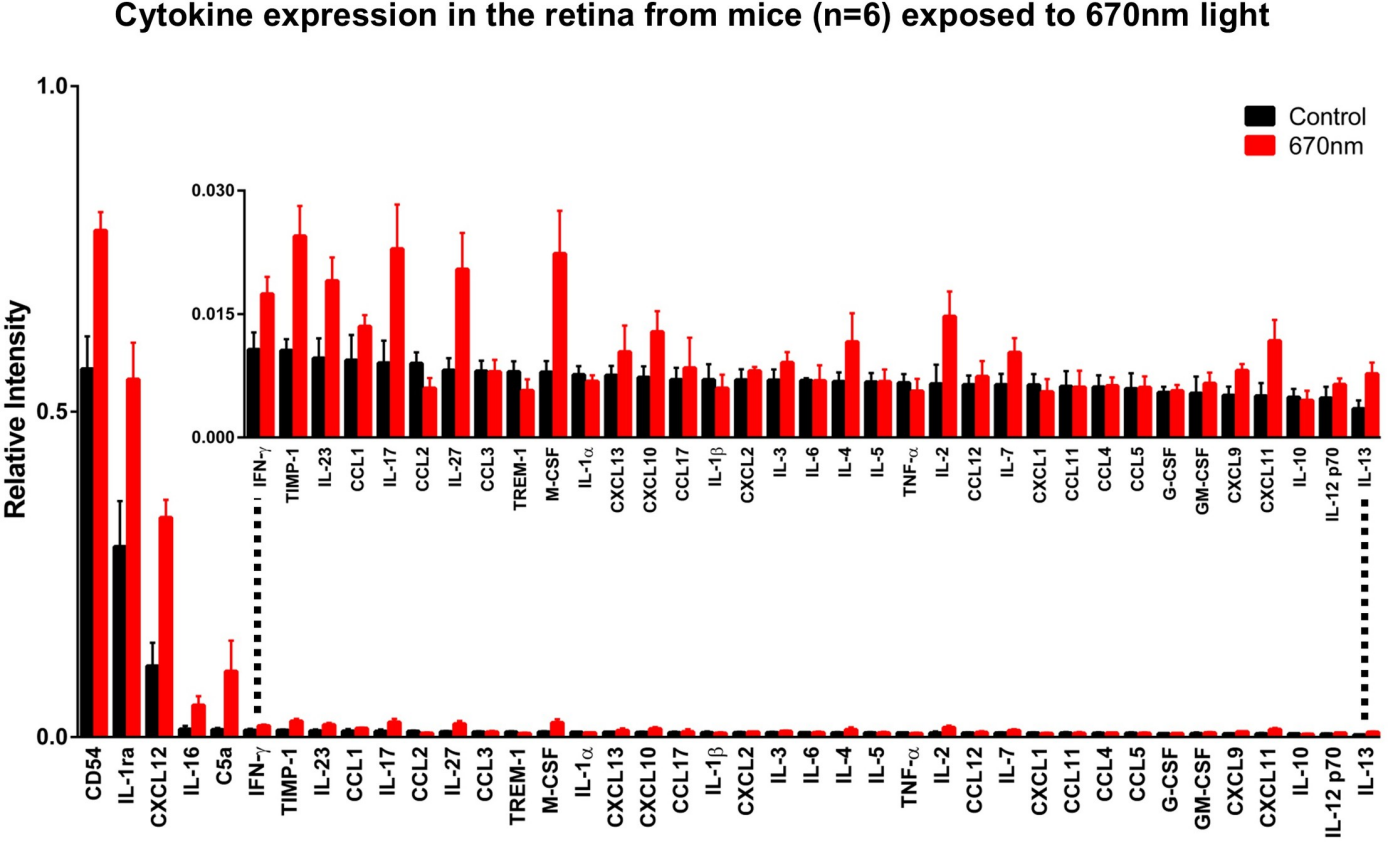
Cytokine expression retina. Cytokine expression from the retina of 12-month-old C57Bl6 mice (N = 6) following exposure with 670nm light (N = 6. Red) for 1 week compared to aged match controls (N = 6. Black). The mice used are the same as those for serum in Fig 1. Cytokines are ordered with the highest expressing on the left and the lowest expressing on the right. The scale was expanded in the inset above. Overall, across all 40 cytokines there was a 40% increase in cytokines from 670nm light exposed animals. A 2-way ANOVA revealed a significance of p≤0.0001. Individual cytokine comparisons are presented in Table 2. Error bars = SEM.

Cytokines from the retina (Fig 2) are ordered similarly according to expression in control mice, with those highest in concentration on the left and those lowest in concentration on the right, the scale was also expanded above. There was a statistically significant (p≤0.0001, 2-way ANOVA) difference and an overall 40% increase in cytokine expression from 670nm light exposed animals. Multiple comparisons of individual cytokines from the retina in Fig 2 are displayed in Table 2. Again, no apparent pattern to their properties was observed, however those that displayed a percentage decrease in the retina were increased in blood serum.

**Table 2:** Individual cytokine comparison in the retina between 12-month-old C57Bl6 mice exposed to 670nm light for 1 week compared to age matched controls, represented in Fig 2.

Target	P-Value	Significance	% Difference
C5a	0.0076	**	89
IL-16	0.0043	**	75
CXCL12	0.0022	**	68
M-CSF	0.0043	**	64
IL-17	0.0206	*	60
IL-27	0.0206	*	60
CXCL11	0.0076	**	57
TIMP-1	0.0022	**	57
IL-2	0.0206	*	56
IL-13	0.0325	*	55
IL-23	0.013	*	49
IL-1ra	0.0076	**	47
CXCL10	0.0898	ns	43
IL-4	0.119	ns	42
IFN-γ	0.0465	*	39
IL-7	0.0898	ns	38
CXCL9	0.026	*	37
CCL2	0.066	ns	-33
CCL1	0.1937	ns	30
TREM-1	0.1937	ns	-29
CD54	0.0043	**	27
CXCL13	>0.9999	ns	27
IL-12 p70	0.237	ns	26
IL-3	0.237	ns	23
GM-CSF	0.237	ns	18
CCL17	0.3939	ns	17
TNF-α	0.3939	ns	-15
IL-1β	0.4491	ns	-15
CXCL2	0.3377	ns	13
CCL12	0.2857	ns	13
CXCL1	0.3377	ns	-13
IL-1α	0.3939	ns	-10
IL-10	0.4491	ns	-8
G-CSF	>0.9999	ns	4
CCL4	0.4491	ns	3
CCL5	0.4491	ns	2
CCL11	>0.9999	ns	2
CCL3	>0.9999	ns	1
IL-5	0.4491	ns	1
IL-6	0.4491	ns	0

The final column represents a heat map of the relative percentage increase or decrease values in expression of each cytokine with red indicating a relatively large change and green a relatively small change. Mann-Whitney U statistical test

*p≤0.05

**p≤0.01, ns = non-significant.

## Discussion

Cytokine expression in mouse serum increases with age [14]. We show here that it also increases with exposure to 670nm light. Aged increases are relatively uniform, and no cytokine was found to decrease in the older age group compared to the young. The increase in cytokine expression was unexpected following 670nm exposure. Increases particularly in serum were marked and specific. While some increased by as much as 5-fold others did not change and some were reduced. That there are such changes in serum underlines the potential for 670nm light exposure to have a systemic impact.

The parameters we have used in terms of the mice, their age and patterns of 670nm exposure, duration and energy, are consistent with our previous studies. This is the key reason why we have focused our analysis on an acute exposure of one week. These have consistently shown improvements in mitochondrial function along with reduced cell death and improved retinal function [9, 15–17]. In the retina they have also shown reduced markers of tissue inflammation and stress using immune staining on tissue sections [7, 18, 19]. But commonly they have not been the same inflammatory markers that have been used here, rather they have often been those associated with complement [7, 8].

In retinae, reductions have been shown in TNF-α and complement proteins in immune staining [18, 19]. The key difference between these results and those presented in is study is that here the cytokines, particularly those in the serum, were unbound. It is possible that an overall less marked increase in cytokine expression in the retina after 670nm exposure compared to serum is because this includes bound and unbound elements, and perhaps the bound elements were reduced. Hence, TNF-α as expressed in tissue section and immune staining is significantly reduced following 670nm exposure [7, 8], but is elevated in serum in this study using identical exposure protocols. It did not appear to change much in the array on the retina after 670nm exposure, but this picture may be blurred by inclusion of bound and unbound elements in retinal samples.

If there is consistency between previous studies and the data presented here, it highlights that the selective cytokines that are up regulated are likely to be protective. There is evidence for the protective impact of cytokines [20, 21] and often different cytokines act in complex synergy [22]. The consequence is that it is very difficult to determine what the impact of up and down regulation of any cytokine is as it does not occur in isolation but is likely to be highly interactive with the dynamics of other cytokines and inflammatory agents. Hence, while those that shift in either direction following 670nm exposure are associated with improved cellular and organ function, determining their complex interactions are beyond the scope of this study.

Our data do offer a potential explanation for enigmatic results that have been described as the abscopal effect when treatment of tissues distal to pathology is found to be effective. Two labs have shown that retinal pathology may be partially ameliorated by long wavelength exposure to regions other than the eye [13, 23], while others have shown similar general systemic effects following focal long wavelength exposure [24, 25]. Our results provide a potential route for this effect via shifts in the patterns of circulating cytokines.

This study has used experimental metrics consistent with those employed previously for consistency. However, there is far less data available for potential interactions between light, mitochondria and cytokines. The relationship between immunity and mitochondrial function is far from clear [26–28]. Consequently, we have little idea of whether this snapshot we provide at one time following a week’s exposure to 670nm light is representative. It is possible that cytokine expression is reduced after 670nm light exposure and that what we are viewing is actually a rebound effect. ATP upregulation following a single 1 min 670nm exposure occurs within an hour and improves respiration significantly for almost a week [3, 11]. But we know no more about the dynamics of this let alone how immunity changes over time. To have used different time points/exposures would have robbed our data set from its relationship to the majority of other 670nm studies undertaken on mice that reveals positive effects. However, to explore the large number of permutations and combinations of lights, energies and potential post exposure time periods would be complex undertaking.

The use of mice represents an additional problem. The impact of long wavelength light appears to be species independent, even having commonality between invertebrates and mammals including humans [4, 10, 17]. However, mouse immunity is fundamentally different from that of humans, which is exacerbated by laboratory mice being maintained in pathogen free environments [29–32]. When cytokine arrays specific for mice and human are used in aged retinae from mice and old world primates in ageing, the patterns of up regulation in the two species have almost no relationship with one another [14]. Hence while our results have a degree of significance, the problematic nature of mouse immunity needs to be kept in mind.

While long wavelength light has shown largely consistent patterns of improvement from cells through to system function in ageing, the fact that it is associated with increased cytokine expression remains an issue. It is possible that this is protective, and that cytokine expression is different at separate time points following exposure. But it is clearly important that the experiments undertaken here, particularly those on serum, are repeated in aged humans where long wavelengths are increasingly being employed in ageing and age related disease.

## Supporting information

S1 File (DOCX)
