# Supplementary material for: Exposure to long wavelength light that improves aged mitochondrial function shifts acute cytokine expression in serum and the retina
Source: PLoS One. 2023 Jul 21;18(7):e0284172. doi: 10.1371/journal.pone.0284172 (PMC10361513; doi:10.1371/journal.pone.0284172)
Supplement: S1 File — (DOCX) [file pone.0284172.s001.docx]

|  | **Control** | **670nm** |
| --- | --- | --- |
| **CD54** | 0.9801266 | 0.9830849 |
| **C5a** | 0.6869197 | 0.7018372 |
| **CXCL12** | 0.6455781 | 0.7770416 |
| **M-CSF** | 0.3580377 | 0.4255498 |
| **CXCL13** | 0.124738 | 0.1947494 |
| **TIMP-1** | 0.0829995 | 0.0957127 |
| **IFN-g** | 0.0168976 | 0.0192854 |
| **IL-17** | 0.0097642 | 0.0106098 |
| **CCL4** | 0.0080988 | 6.019E-05 |
| **IL-7** | 0.0066748 | 0.0156876 |
| **CXCL11** | 0.0065059 | 0.008722 |
| **CXCL10** | 0.0063691 | 0.0127219 |
| **CXCL9** | 0.0060098 | 0.0156028 |
| **TREM-1** | 0.0058381 | 0.0054089 |
| **IL-12 p70** | 0.0055565 | 0 |
| **IL-1b** | 0.0045831 | 0.0032065 |
| **TNF-a** | 0.0040521 | 0.0143689 |
| **IL-16** | 0.0033414 | 0.0223468 |
| **CXCL2** | 0.0032315 | 0 |
| **CCL2** | 0.0027139 | 0.0025936 |
| **IL-27** | 0.0026844 | 0 |
| **IL-1a** | 0.0020891 | 0.0058138 |
| **IL-1ra** | 0.001778 | 0.0160844 |
| **IL-3** | 0.00166 | 0.0001094 |
| **CCL1** | 0.0012926 | 0.0099012 |
| **CCL3** | 0.0012577 | 0 |
| **CCL12** | 0.0007294 | 0 |
| **IL-4** | 0.0003218 | 0 |
| **IL-13** | 0 | 0.0122076 |
| **G-CSF** | 0 | 0.0086865 |
| **IL-23** | 0 | 0.0052228 |
| **CCL17** | 0 | 0.0035649 |
| **IL-6** | 0 | 0.0027988 |
| **CXCL1** | 0 | 0.0015075 |
| **IL-2** | 0 | 6.019E-05 |
| **CCL5** | 0 | 0 |
| **CCL11** | 0 | 0 |
| **GM-CSF** | 0 | 0 |
| **IL-10** | 0 | 0 |
| **IL-5** | 0 | 0 |

**Table 1**: Cytokine signal values pooled from the above data for the blood serum of 12-month-old C57Bl6 mice following exposure with 670nm light. Cytokine levels were assessed using the Proteome Profiler Mouse Cytokine Array Panel A following manufacturers instructions (R&D Systems, Minneapolis, USA). Protein Array Analyzer for Image J was used to quantify and determine each cytokine spot pixel density from the X-ray film. Signal (pixel density) values represented here, were averaged for a pair of duplicate spots representing each cytokine and background signal values were subtracted from the cytokine values.

|  | **Control** | **670nm** |
| --- | --- | --- |
| **CD54** | 0.7865859 | 0.8168989 |
| **CXCL12** | 0.1083552 | 0.0613829 |
| **IL-1ra** | 0.0575042 | 0.0419805 |
| **C5a** | 0.0257725 | 0.0261456 |
| **IL-7** | 0.0179133 | 0.0195031 |
| **CXCL11** | 0.0169522 | 0.0157941 |
| **IFN-g** | 0.0165556 | 0.020695 |
| **TIMP-1** | 0.0127747 | 0.0113231 |
| **IL-17** | 0.0114034 | 0.0183629 |
| **IL-16** | 0.0057967 | 0.006667 |
| **M-CSF** | 0.0057141 | 0 |
| **IL-1a** | 0.0045905 | 0 |
| **CCL17** | 0.0043923 | 0.0010232 |
| **CXCL10** | 0.0041196 | 0 |
| **CCL2** | 0.0036432 | 0.0024818 |
| **IL-4** | 0.0035937 | 0.0036138 |
| **CXCL2** | 0.00331 | 0 |
| **TREM-1** | 0.002908 | 0.0023756 |
| **CCL1** | 0.0026354 | 0.0040302 |
| **IL-13** | 0.002572 | 0.0060738 |
| **TNF-a** | 0.0023132 | 0.0029988 |
| **IL-1b** | 0.0022085 | 0.0002095 |
| **IL-5** | 0.0020818 | 0.0039948 |
| **CXCL9** | 0.0017597 | 0.0053935 |
| **CXCL1** | 0.0014237 | 0 |
| **IL-6** | 0.0013659 | 0.0053935 |
| **IL-23** | 0.0009969 | 0 |
| **CXCL13** | 0.0008894 | 0.0024627 |
| **CCL11** | 0.000369 | 0.002509 |
| **CCL4** | 6.609E-05 | 0.0013688 |
| **IL-10** | 3.029E-05 | 0.0045118 |
| **GM-CSF** | 0 | 0.0015348 |
| **CCL5** | 0 | 0.0042479 |
| **G-CSF** | 0 | 0.0041063 |
| **IL-12 p70** | 0 | 0.0007238 |
| **IL-3** | 0 | 0.0006749 |
| **IL-27** | 0 | 0 |
| **CCL3** | 0 | 0 |
| **CCL12** | 0 | 0 |
| **IL-2** | 0 | 0 |

**Table 2**: Cytokine signal values pooled from the retina of 12-month-old C57Bl6 mice following exposure with 670nm light. Cytokine levels were assessed using the Proteome Profiler Mouse Cytokine Array Panel A following manufacturers instructions (R&D Systems, Minneapolis, USA). Protein Array Analyzer for Image J was used to quantify and determine each cytokine spot pixel density from the X-ray film. Signal (pixel density) values represented here, were averaged for a pair of duplicate spots representing each cytokine and background signal values were subtracted from the cytokine values.

|  | **Control** | | | | | | **670nm** | | | | | |
| --- | --- | --- | --- | --- | --- | --- | --- | --- | --- | --- | --- | --- |
| **CXCL12** | 0.909488 | 0.941942 | 1.02767 | 0.970733 | 1.026017 | 0.944512 | 1.012705 | 0.934111 | 0.990194 | 0.939291 | 0.952504 | 1.014761 |
| **CD54** | 0.8003 | 0.882141 | 0.886057 | 0.866464 | 0.892799 | 0.961879 | 0.866948 | 0.935631 | 0.790953 | 0.843668 | 0.776383 | 0.820754 |
| **C5a** | 0.798357 | 0.854174 | 0.962589 | 0.704672 | 0.827874 | 0.75009 | 0.916255 | 0.745132 | 0.81412 | 0.754279 | 0.815989 | 0.80801 |
| **M-CSF** | 0.695914 | 0.709829 | 0.751682 | 0.645043 | 0.720802 | 0.722304 | 0.730958 | 0.678636 | 0.701984 | 0.684945 | 0.613767 | 0.732718 |
| **TIMP-1** | 0.665616 | 0.63318 | 0.728967 | 0.626774 | 0.773964 | 0.518089 | 0.816468 | 0.866435 | 0.820688 | 0.6858 | 0.650695 | 0.616171 |
| **CXCL13** | 0.608835 | 0.493216 | 0.662999 | 0.943657 | 0.642283 | 0.526666 | 0.608023 | 0.693661 | 0.765786 | 0.70314 | 0.780564 | 0.647556 |
| **IL-16** | 0.37166 | 0.324044 | 0.48453 | 0.288897 | 0.385525 | 0.298461 | 0.482819 | 0.433554 | 0.526053 | 0.261473 | 0.667025 | 0.447491 |
| **IL-1ra** | 0.280189 | 0.244678 | 0.512397 | 0.336934 | 0.301861 | 0.188317 | 0.74382 | 0.420297 | 0.577252 | 0.240853 | 0.492688 | 0.276542 |
| **CXCL1** | 0.28322 | 0.112338 | 0.401396 | 0.099378 | 0.220737 | 0.052559 | 0.316724 | 0.241297 | 0.182735 | 0.182634 | 0.638608 | 0.164681 |
| **CCL2** | 0.197839 | 0.084371 | 0.245363 | 0.11412 | 0.124158 | 0.049596 | 0.338752 | 0.241129 | 0.316754 | 0.180789 | 0.416579 | 0.14734 |
| **IL-13** | 0.260248 | 0.06542 | 0.16108 | 0.248892 | 0.02759 | 0.032735 | 0.147602 | 0.052885 | 0.100388 | 0.061707 | 0.223666 | 0.095486 |
| **CXCL10** | 0.15507 | 0.078858 | 0.182025 | 0.085265 | 0.074626 | 0.079575 | 0.252419 | 0.199267 | 0.262897 | 0.169904 | 0.18876 | 0.099038 |
| **CXCL9** | 0.170442 | 0.041299 | 0.160789 | 0.102973 | 0.050534 | 0.019691 | 0.385507 | 0.148476 | 0.237842 | 0.158324 | 0.234928 | 0.099716 |
| **IFN-g** | 0.112215 | 0.044249 | 0.15831 | 0.061809 | 0.08517 | 0.073737 | 0.233911 | 0.140793 | 0.213503 | 0.136908 | 0.147609 | 0.132561 |
| **IL-27** | 0.068339 | 0.037593 | 0.080232 | 0.043485 | 0.071615 | 0.078932 | 0.159323 | 0.071829 | 0.167131 | 0.06909 | 0.045192 | 0.214182 |
| **TNF-a** | 0.093146 | 0.010932 | 0.150783 | 0.062344 | 0.021052 | 0.029933 | 0.163777 | 0.107486 | 0.141504 | 0.107473 | 0.152069 | 0.034212 |
| **CCL12** | 0.076586 | 0.019974 | 0.125416 | 0.056313 | 0.02809 | 0.034207 | 0.15412 | 0.045245 | 0.107746 | 0.03746 | 0.14122 | 0.076263 |
| **TREM-1** | 0.069878 | 0.012088 | 0.126071 | 0.042352 | 0.043869 | 0.0267 | 0.142887 | 0.143563 | 0.175463 | 0.053634 | 0.089945 | 0.059061 |
| **IL-17** | 0.060651 | 0.007542 | 0.085701 | 0.057561 | 0.04608 | 0.053635 | 0.149944 | 0.077579 | 0.139933 | 0.097034 | 0.013426 | 0.122316 |
| **IL-2** | 0.028847 | 0.040917 | 0.075299 | 0.009209 | 0.059041 | 0.0916 | 0.093612 | 0.020618 | 0.051591 | 0.011916 | 0.047391 | 0.120695 |
| **IL-23** | 0.051383 | 0.035509 | 0.061437 | 0.049841 | 0.044901 | 0.060331 | 0.127792 | 0.049723 | 0.094493 | 0.046216 | 0.00815 | 0.108073 |
| **CCL1** | 0.036484 | 0.026327 | 0.100974 | 0.019123 | 0.055437 | 0.049431 | 0.144757 | 0.030825 | 0.088563 | 0.009717 | 0.125808 | 0.097042 |
| **IL-4** | 0.062123 | 0.008893 | 0.116234 | 0.035394 | 0.014987 | 0.033933 | 0.08389 | 0.03141 | 0.065717 | 0.043114 | 0.087548 | 0.063648 |
| **G-CSF** | 0.081747 | 0.015913 | 0.092307 | 0.017318 | 0.013298 | 0.030861 | 0.099742 | 0.096687 | 0.090443 | 0.03582 | 0.095092 | 0.064544 |
| **IL-7** | 0.065868 | 0.009605 | 0.102959 | 0.019795 | 0.023027 | 0.020345 | 0.161669 | 0.055511 | 0.126654 | 0.052162 | 0.090029 | 0.044211 |
| **IL-1a** | 0.043883 | 0 | 0.126092 | 0.027396 | 0.018298 | 0.020281 | 0.227808 | 0.10203 | 0.1712 | 0.108756 | 0.16777 | 0.037361 |
| **CXCL2** | 0.04923 | 0.008296 | 0.080274 | 0.028788 | 0.035178 | 0.020341 | 0.136974 | 0.047209 | 0.112631 | 0.055256 | 0.042465 | 0.07229 |
| **CCL4** | 0.051786 | 0.001596 | 0.106397 | 0.030418 | 0.014047 | 0.017287 | 0.143431 | 0.037408 | 0.078595 | 0.058526 | 0.108413 | 0.052025 |
| **CCL5** | 0.048438 | 0.008811 | 0.062083 | 0.029649 | 0.013475 | 0.022816 | 0.111806 | 0.060291 | 0.105796 | 0.058252 | 0.021987 | 0.057097 |
| **CCL3** | 0.043332 | 0 | 0.099952 | 0.018402 | 0.006148 | 0.017357 | 0.102572 | 0.021468 | 0.062081 | 0.016116 | 0.067194 | 0.042829 |
| **CXCL11** | 0.029802 | 0.011723 | 0.060068 | 0.02363 | 0.017549 | 0.032932 | 0.08181 | 0.037607 | 0.044508 | 0.029007 | 0.072136 | 0.036875 |
| **IL-3** | 0.044305 | 0.00222 | 0.060377 | 0.014685 | 0.010924 | 0.027167 | 0.082302 | 0.037474 | 0.079902 | 0.031027 | 0.048469 | 0.02125 |
| **CCL11** | 0.047702 | 0 | 0.068587 | 0.026296 | 0.012578 | 0.00316 | 0.123112 | 0.023913 | 0.098725 | 0.040265 | 0.063712 | 0.008881 |
| **IL-1b** | 0.034697 | 0.007406 | 0.057205 | 0.015231 | 0.015697 | 0.02606 | 0.112064 | 0.044439 | 0.09023 | 0.048345 | 0.000901 | 0.053428 |
| **GM-CSF** | 0.042011 | 0 | 0.080485 | 0.015462 | 0.002612 | 0.01219 | 0.095925 | 0.035195 | 0.044473 | 0.026238 | 0.070306 | 0.017559 |
| **CCL17** | 0.029143 | 0 | 0.074027 | 0.011901 | 0.004396 | 0.009719 | 0.083785 | 0.026007 | 0.05672 | 0.047921 | 0.068963 | 0.007168 |
| **IL-10** | 0.027075 | 0.002383 | 0.078386 | 0.008956 | 0.008467 | 0.002538 | 0.096635 | 0.023317 | 0.068278 | 0.016836 | 0.099024 | 0.030203 |
| **IL-12 p70** | 0.024234 | 0 | 0.06607 | 0.008811 | 0.006126 | 0.00845 | 0.109888 | 0.017837 | 0.052152 | 0.005614 | 0.083584 | 0.011926 |
| **IL-6** | 0.011055 | 0 | 0.045134 | 0.007051 | 0.002012 | 0.002629 | 0.046175 | 0.004493 | 0.012115 | 0.000628 | 0.075775 | 0.007921 |
| **IL-5** | 0.010319 | 0 | 0.023901 | 0.000186 | 0.000767 | 0.00522 | 0.029597 | 0.009549 | 0.010928 | 0.000851 | 0.026136 | 0 |

**Table 3**: Cytokine signal valuesfrom blood serum of 12-month-old C57Bl6 mice (N=6) following exposure with 670nm light (N=6). Cytokine levels were assessed using the Proteome Profiler Mouse Cytokine Array Panel A following manufacturers instructions (R&D Systems, Minneapolis, USA). Protein Array Analyzer for Image J was used to quantify and determine each cytokine spot pixel density from the X-ray film. Signal (pixel density) values represented here, were averaged for a pair of duplicate spots representing each cytokine and background signal values were subtracted from the cytokine values.

|  | **Control** | | | | | | **670nm** | | | | | |
| --- | --- | --- | --- | --- | --- | --- | --- | --- | --- | --- | --- | --- |
| **CD54** | 0.5208794 | 0.7067544 | 0.6367505 | 0.0864397 | 0.5505946 | 0.4132787 | 0.7997921 | 0.8404244 | 0.8650976 | 0.6866204 | 0.7545796 | 0.7243103 |
| **IL-1ra** | 0.392617 | 0.2934946 | 0.432039 | 0.0109793 | 0.4496345 | 0.1780181 | 0.6029512 | 0.7631693 | 0.6225328 | 0.4757999 | 0.4382504 | 0.3955287 |
| **CXCL12** | 0.2717102 | 0.1009599 | 0.1198133 | 0.0357935 | 0.0978385 | 0.0286774 | 0.3327033 | 0.4206694 | 0.3811952 | 0.3644655 | 0.2921426 | 0.2326762 |
| **IL-16** | 0.0370835 | 0.0045728 | 0.0034961 | 0.0146328 | 0.0061326 | 0.0081503 | 0.0423806 | 0.1162273 | 0.0469609 | 0.028455 | 0.0383073 | 0.0222038 |
| **C5a** | 0.0047 | 0.0055169 | 0.0092226 | 0.0124779 | 0.0163467 | 0.0206597 | 0.0428557 | 0.2241027 | 0.2717558 | 0.0308812 | 0.0279908 | 0.0096692 |
| **IFN-g** | 0.0195165 | 0.0115517 | 0.0056321 | 0.0089474 | 0.0122963 | 0.006083 | 0.0105074 | 0.0260212 | 0.0185752 | 0.016638 | 0.0151858 | 0.017743 |
| **TIMP-1** | 0.0135414 | 0.0119709 | 0.0051707 | 0.0139766 | 0.0103764 | 0.0083985 | 0.0271704 | 0.0471467 | 0.0358534 | 0.0263185 | 0.0167312 | 0.0163514 |
| **IL-23** | 0.0198783 | 0.005834 | 0.0093904 | 0.0097042 | 0.0022721 | 0.0106971 | 0.0133614 | 0.0317598 | 0.0211132 | 0.0145365 | 0.0194147 | 0.0139905 |
| **CCL1** | 0.0194146 | 0.0008455 | 0.0054259 | 0.0179396 | 0.0070338 | 0.0056988 | 0.0095881 | 0.0144214 | 0.0146496 | 0.0091848 | 0.0173577 | 0.015822 |
| **IL-17** | 0.0144477 | 0.0061686 | 0.0082891 | 0.0188232 | 0.0065884 | 0 | 0.0096949 | 0.0455124 | 0.0256036 | 0.0282281 | 0.0155721 | 0.0128815 |
| **CCL2** | 0.0094913 | 0.0071586 | 0.0062509 | 0.0114304 | 0.005587 | 0.0140565 | 0.0103042 | 0.0090581 | 0.0033608 | 0.0059766 | 0.0031638 | 0.0040888 |
| **IL-27** | 0.0125719 | 0.0066936 | 0.0098064 | 0.0096409 | 0.0019061 | 0.0083237 | 0.0162086 | 0.0389005 | 0.0231248 | 0.0192737 | 0.0193242 | 0.0058165 |
| **CCL3** | 0.0125825 | 0.0054746 | 0.0059608 | 0.0068075 | 0.0061257 | 0.0114587 | 0.0084796 | 0.0060695 | 0.0058257 | 0.003299 | 0.0115869 | 0.0126025 |
| **TREM-1** | 0.0063123 | 0.0045587 | 0.007576 | 0.0058979 | 0.0121444 | 0.0114383 | 0.0074157 | 0.0040054 | 0.0093608 | 0.0080083 | 0.0053879 | 0 |
| **M-CSF** | 0.0136503 | 0.0071374 | 0.0070411 | 0.0044625 | 0.0057044 | 0.0097076 | 0.0256969 | 0.0734408 | 0.0412886 | 0.0157304 | 0.0126345 | 0.0163586 |
| **IL-1a** | 0.0052374 | 0.0058304 | 0.0089954 | 0.0063079 | 0.0121478 | 0.0072493 | 0.0099944 | 0.00682 | 0.0046961 | 0.0059486 | 0.0072013 | 0.0063102 |
| **CXCL13** | 0.0120239 | 0.0047348 | 0.0056706 | 0.0061029 | 0.0098239 | 0.0070044 | 0.0119912 | 0.0247998 | 0.004215 | 0.0114469 | 0.0041245 | 0.0058058 |
| **CXCL10** | 0.009003 | 0.0042416 | 0.0045624 | 0.0094843 | 0.0045028 | 0.0120436 | 0.0078805 | 0.0238412 | 0.0151028 | 0.0133181 | 0.0081898 | 0.0086783 |
| **CCL17** | 0.005406 | 0.0097867 | 0.0025591 | 0.0051373 | 0.0068405 | 0.0124516 | 0.0015114 | 0.0031661 | 0.0188471 | 0.0064199 | 0 | 0.0206727 |
| **IL-1b** | 0.0129443 | 0.0044495 | 0.0019997 | 0.0112999 | 0.0027866 | 0.0086535 | 0.0044791 | 0.0120025 | 0.0053027 | 0 | 0.0054958 | 0.0085817 |
| **CXCL2** | 0.0038921 | 0.0080146 | 0.0073033 | 0.0092084 | 0.0025656 | 0.0109725 | 0.0085174 | 0.0087033 | 0.0077675 | 0.0061406 | 0.0097491 | 0.0075944 |
| **IL-3** | 0.0062877 | 0.0078984 | 0.0085234 | 0.0017037 | 0.0111326 | 0.0063652 | 0.0109549 | 0.0135753 | 0.0096815 | 0.0093628 | 0.0054715 | 0.005559 |
| **IL-6** | 0.005666 | 0.0073876 | 0.0075375 | 0.0073294 | 0.0066782 | 0.0068616 | 0.0007402 | 0.0061172 | 0.0130807 | 0.0030651 | 0.0107585 | 0.007598 |
| **IL-4** | 0.008455 | 0.0024484 | 0.0087926 | 0.0071542 | 0.0094475 | 0.0044543 | 0.0104488 | 0.0269356 | 0.0115049 | 0.013168 | 0.0055237 | 0.0020855 |
| **IL-5** | 0.0079106 | 0.0034172 | 0.0070655 | 0.0076426 | 0.0105732 | 0.003866 | 0.0079769 | 0.0051483 | 0.0029738 | 0.003676 | 0.0086214 | 0.0124344 |
| **TNF-a** | 0.0047667 | 0.0027303 | 0.0068872 | 0.0072959 | 0.007721 | 0.0104319 | 0.0099152 | 0.0070725 | 0 | 0.0028207 | 0.0057325 | 0.0082276 |
| **IL-2** | 0.0086693 | 0.0052492 | 0.003587 | 0.0167951 | 0 | 0.0047433 | 0.0117778 | 0.0139233 | 0.0202451 | 0.0054215 | 0.0261775 | 0.0107996 |
| **CCL12** | 0.0044822 | 0.0039421 | 0.0063209 | 0.0056928 | 0.0065504 | 0.0115267 | 0.0042931 | 0.0074853 | 0.0101557 | 0.0090172 | 0.0003063 | 0.0132321 |
| **IL-7** | 0.0090522 | 0.0004721 | 0.0060132 | 0.0061737 | 0.0076796 | 0.0091126 | 0.0073056 | 0.017045 | 0.0138895 | 0.0101029 | 0.0071735 | 0.0064676 |
| **CXCL1** | 0.0020128 | 0.0070318 | 0.0072858 | 0.0037505 | 0.0067472 | 0.0114893 | 0 | 0.0050323 | 0.0033469 | 0.0074114 | 0.0061502 | 0.0113433 |
| **CCL11** | 0.005687 | 0.0003452 | 0.0142814 | 0.0046713 | 0.0056457 | 0.006661 | 0.0058424 | 0.0038791 | 0.0022835 | 0.0079804 | 0.0017681 | 0.0149778 |
| **CCL4** | 0.0052163 | 0.0075919 | 0.0056846 | 0 | 0.0085946 | 0.0097246 | 0.0061316 | 0.0055338 | 0.0078373 | 0.0052155 | 0.0028924 | 0.01017 |
| **CCL5** | 0.0084164 | 0.0069895 | 0.0008565 | 0.0056704 | 0.0008874 | 0.0127916 | 0.0077463 | 0.0058682 | 0.0043753 | 0.0110839 | 0.0061711 | 0.0012771 |
| **G-CSF** | 0.0060032 | 0.0053231 | 0.0027794 | 0.0070685 | 0.0043716 | 0.0072833 | 0.0042312 | 0.006165 | 0.0027681 | 0.0068074 | 0.0070899 | 0.0069684 |
| **GM-CSF** | 0.0043593 | 0 | 0.0052721 | 0.0147931 | 0.0038709 | 0.0039986 | 0.0101355 | 0.0039849 | 0.0018896 | 0.00561 | 0.0087502 | 0.0090074 |
| **CXCL9** | 0.0059189 | 0.0038224 | 0.0007621 | 0.0051336 | 0.006509 | 0.0085515 | 0.0090098 | 0.0098531 | 0.0045636 | 0.0064269 | 0.0093558 | 0.0060848 |
| **CXCL11** | 0 | 0.0036709 | 0.0056601 | 0.005402 | 0.0038191 | 0.0116933 | 0.0072987 | 0.0229132 | 0.0122579 | 0.0138627 | 0.0066966 | 0.0075372 |
| **IL-10** | 0.0057503 | 0.0020539 | 0.0037722 | 0.0040785 | 0.0044061 | 0.0092928 | 0.0067616 | 0 | 0.0046368 | 0.0044615 | 0.0031186 | 0.0081167 |
| **IL-12 p70** | 0.0068673 | 0.0046186 | 0 | 0.0067068 | 0.0017921 | 0.0087929 | 0.0058458 | 0.0092731 | 0.006115 | 0.0078373 | 0.0048276 | 0.0047577 |
| **IL-13** | 0.0053393 | 0.0006694 | 0.0005559 | 0.0057189 | 0.0030076 | 0.0057328 | 0.0044687 | 0.0084099 | 0.011341 | 0.0075754 | 0.0112423 | 0.003384 |

**Table 4**: Cytokine signal values from retina of 12-month-old C57Bl6 mice (N=6) following exposure with 670nm light (N=6). Cytokine levels were assessed using the Proteome Profiler Mouse Cytokine Array Panel A following manufacturers instructions (R&D Systems, Minneapolis, USA). Protein Array Analyzer for Image J was used to quantify and determine each cytokine spot pixel density from the X-ray film. Signal (pixel density) values represented here, were averaged for a pair of duplicate spots representing each cytokine and background signal values were subtracted from the cytokine values.
